# The effect of concomitant fibromyalgia in HIV infected patients receiving antiretroviral therapy: a prospective cross-sectional study

**DOI:** 10.1186/s12941-019-0330-0

**Published:** 2019-10-31

**Authors:** Umit Secil Demirdal, Neriman Bilir, Tuna Demirdal

**Affiliations:** 1 Department of Physical Medicine and Rehabilitation, Ataturk Training and Research Hospital, Faculty of Medicine, Izmir Katip Celebi University, Izmir, Turkey; 2 Department of Clinical Microbiology and Infectious Diseases, Ataturk Training and Research Hospital, Faculty of Medicine, Izmir Katip Celebi University, Basın Sitesi, 35360 Karabaglar/Izmir, Turkey

**Keywords:** HIV, Antiretroviral therapy, Fibromyalgia, Depression, Fatigue, Sleep, Quality of life

## Abstract

**Background:**

HIV infected patients receiving antiretroviral therapy (ART) have extensive musculoskeletal system involvement. Arthralgia and myalgia are the most common forms. Fibromyalgia Syndrome (FMS) is a chronic pain syndrome of the musculoskeletal system characterized by diffuse pain including arthralgia and myalgia. These overlapping symptoms are suggested the relationship between HIV and FMS. The primary purpose of this study was to determine the prevalence of FMS in HIV/AIDS patients. The secondary objective was to investigate the effects of FMS on functional status, depression, fatigue, sleep pattern and quality of life.

**Methods:**

A total of 225 HIV infected patients who were receiving ART were included in this cross-sectional prospective study. The demographic data of the participants, CD4 T-lymphocyte count (cells/mm^3^), viral load (> 40 copy/ml), and ART regimens were recorded. FMS diagnosis was based on 2016 revision of diagnostic criteria. All patients completed the following questionnaires: Fibromyalgia Impact Questionnaire (FIQ), Beck Depression Inventory (BDI), Pittsburgh Sleep Quality Index (PSQI), Fatigue Severity Scale (FSS), and SF-36 scale.

**Results:**

FMS was found in 20% of the HIV infected patients (n = 45). The mean duration of disease was 4.74 ± 4.42 years; it was significantly longer in patients with FMS (p = 0.007). The median CD4 T-lymphocyte count was found to be 616.00 ± 303.91 cells/mm^3^, and it was significantly higher in patients without FMS (p = 0.06). No statistically significant difference was found between the two groups according to the drug regimens used. A statistically significant difference was found in FIQ, BDI, PSQI, FSS and all subgroups of the SF-36 scale between the patients with and without FMS (p = 0.001).

**Conclusions:**

A slightly higher frequency of FMS was determined in HIV infected patients receiving ART compared to previous studies. It was shown that presence of FMS negatively affected the function, depression, fatigue, sleep, and quality of life. Detection of FMS may decrease depression, fatigue, and sleep disorders and increase the quality of life in HIV infected patients. FMS should be distinguished correctly for an accurate treatment management of HIV and for increasing ART compliance.

## Introduction

Fibromyalgia Syndrome (FMS) is a chronic pain syndrome of the musculoskeletal system characterized by generalized pain. This syndrome is a central nervous system disease characterized by central sensitization which is described as increased painful response to non-painful stimulus. Patients may have chronic fatigue, weakness, concentration and memory problems, and sleep disorders as well as generalized pain. In addition, FMS accompanying other disorders causes exacerbation of symptoms of the primary disease and decrease in quality of life. Although FMS was described as an illness by the World Health Organization (WHO) in 1992, there are still questions in terms of etiological factors, diagnostic methods and treatment options [1, 2].

Human immunodeficiency virus (HIV) is a global public health problem that leads to Acquired Immune Deficiency Syndrome (AIDS). Numerous opportunistic infections and cancer may be observed in HIV/AIDS patients as a result of progressive destruction in CD4 T lymphocytes and impaired cellular immunity [3]. HIV infection, which previously had a high mortality rate because of these devastating damages, has currently been considered a chronic disease due to the management of successful antiretroviral therapies (ART). Clinicians are now have to manage a chronic disease process with comorbidities such as neurological, renal, metabolic and musculoskeletal system disorders in HIV infected patients receiving ART [4].

From 1980s when HIV infection was detected for the first time, an enormously wide spectrum of HIV-related musculoskeletal involvement has been shown in many clinical studies [5–7]. Some changes occurred in musculoskeletal involvement after the introduction of ART. For example, the frequency of inflammatory arthritis including reactive arthritis or spondyloarthritis and connective tissue diseases decreased, whereas the frequency of non-inflammatory involvement including osteoporosis and avascular necrosis, increased. Arthralgia and myalgia are the major complaints that have been observed commonly both before and after ART [8].

The presence of overlapping symptoms such as arthralgia and myalgia suggested the relationship between HIV and FMS. In a limited number of studies, the prevalence of FMS was investigated in HIV/AIDS patients on ART [9–11]. Because of the heterogeneity of the studies, the prevalence of FMS ranges between 1 and 17%. FMS is mainly manifested with arthralgia, myalgia and depression in HIV infected patients [8].

According to the publications that reached in the literature, there is no study in which current diagnostic criteria for FMS were used and investigated the effect of FMS on HIV/AIDS patients (PubMed, Google Scholar, Cochrane Database of Systematic Reviews (CDSR), and Web of Science indexes were scanned). In our study, we aimed to determine the prevalence of FMS by using the most up-to-date diagnostic criteria and to investigate the effects of FMS on functional status, depression and fatigue levels, sleep pattern and quality of life in HIV infected patients receiving ART.

## Methods

The protocol of the study which had a prospective cross-sectional design was approved by local Clinical Researches Ethics Committee. The study was conducted in accordance with 1964 Helsinki Declaration. The patients were informed about the study and informed consent was obtained from all patients.

### Selection of the patient group

HIV/AIDS patients aged above 18 years, who had been followed up and treated in the outpatient clinic of infectious diseases, and had receiving ART, were included in the study. The diagnosis of HIV was made by detecting anti-HIV antibody with the enzyme-linked immunosorbent assay (ELISA) and confirming the diagnosis with the western blot method.

The study exclusion criteria included presence of other severe chronic systemic diseases (heart failure, renal failure and similar), presence of severe psychiatric disorder, presence of endocrinopathy, including uncontrolled hypothyroidism or diabetes mellitus, known history of rheumatic diseases, use of antidepressant and similar drugs, use of drugs for the treatment of FMS (gabapentin and pregabalin), recent history of joint and/or muscle trauma (last 1 month), hepatitis B virus (HBV), hepatitis C virus (HCV) and hepatitis D (HDV) positivity, and a diagnosis of malignancy.

The demographic data of the participants (sex, age, occupation), duration of disease, presence of comorbidity (controlled hypertension, hyperlipidemia, diabetes mellitus), CD4 T-lymphocyte count (cells/mm^3^), viral load (> 40 copy/ml), and ART regimens were recorded.

### FMS investigation and making a diagnosis of FMS

For making a diagnosis of FMS, all HIV/AIDS patients was evaluated by the same physical medicine and rehabilitation physician. In the diagnosis of FMS, the revised version of the FMS 2010/2011 diagnostic criteria of American College of Rheumatology (ACR) in 2016 was taken into consideration [12]. Accordingly, the following criteria were used for the diagnosis of FMS: widespread pain in at least four of the five regions defined (excluding jaw pain, chest pain and abdominal pain), continuance of symptoms for at least 3 months with a similar severity, a widespread pain index (WPI) of ≥ 7 and a symptom severity score (SSS) of ≥ 5 or WPI = 4–6 and SSS ≥ 9, and the fact that’’presence of another clinical diagnosis cannot exclude the diagnosis of FMS’’.

### Assessment scales

*Fibromyalgia Impact Questionnaire* (FIQ), which is a specific scale that evaluates physical function and health status in patients with FMS, was used to evaluate functional status in HIV/AIDS patients [13]. Higher scores were considered the increased disease impact which meant low functional level. Validity and reliability of the Turkish version of this questionnaire were previously shown [14]. Presence and severity of depression in the patients was evaluated using the *Beck Depression Inventory* (BDI) [15]. A total score of 0–12 was considered minimal depression, 13–18 was considered mild depression, 19–28 was considered moderate depression and 29–63 was considered severe depression. Validity and reliability of the Turkish version of this inventory were shown [16]. *Pittsburgh Sleep Quality Index* (PSQI), for which validity and reliability study [17] was performed for the Turkish version, was used to evaluate sleep quality. A total score above 5 was interpreted as poor sleep quality [18]. The Turkish version of the *Fatigue Severity Scale* (FSS) was used to evaluate fatigue levels. A high score, especially ≥ 4, was considered increased fatigue [19, 20].

Quality of life was evaluated using the *SF*-*36* scale. With this scale, eight components of health were examined in a total of 36 items: physical function (PF) (limitation in physical activity because of health problems), physical role (PR) (limitation in daily activities because of health problems), emotional role (ER) (limitation in daily activities because of psychological health problems), vitality (V), mental health (MH), social function (SF), bodily pain (BP) and general health (GH) (individual’s evaluation of own general health). Higher scores were considered a better health level and lower scores were considered impairment in health status [21].

### Statistical analysis

The sample size was calculated by using OpenEpi software. Based on a previous research written by Dotan et al. [9] we assumed that the prevalence of FMS would be 14% ± 3.0 with and with confidence limit: 80% sample size was calculated as 221. Statistical analysis was performed using SPSS version 24 software. HIV/AIDS patients were divided into two groups as with FMS and without FMS. The numerical variables were compared using Mann–Whitney U and student t- tests between the two groups. The percentages were compared using Chi square and Fisher exact test. The descriptive analyses were given as mean values and standard deviation. Suitability of assessment scales for normal distribution was determined by Kolmogorov–Smirnov/Shapiro–Wilk tests. As the variables were normally distributed, correlation coefficients and statistical significance were calculated by Pearson test. A p value below 0.05 was considered significant.

## Results

Two hundred thirty-one patients, who presented to our outpatient clinic between June 2018 and June 2019, were included in the study. Seven of the patients were excluded from the study because they were not volunteers. A total of 225 HIV/AIDS patients, who signed informed consent forms, were participated in the study. FMS was found in 20% of the patients (n = 45). The mean duration of disease was 4.74 ± 4.42 years; it was significantly longer in the with FMS patients (p = 0.007). Comorbidity was present in a total of 36 (16%) patients. Comorbidity was found with a statistically significantly higher rate in the patients without FMS (p = 0.029). Since controlled hypertension, hyperlipidemia, and diabetes mellitus were accepted as comorbidities, higher incidence in patients without FMS was not considered clinically significant. The median CD4 T-lymphocyte count was found to be 616.00 ± 303.91 cells/mm^3^, and it was significantly higher in patients without FMS (p = 0.06).

HIV/AIDS patients were divided into six groups according to the treatment regimen: TDF (tenofovir disoproxil fumarate) + FTC (emtricitabine) + RAL (raltegravir); TDF + FTC + DTG (dolutegravir); ABC (abacavir) + 3TC (lamivudine) + DTG; TDF + FTC + DRV/r (darunavir/ritonavir); TDF + FTC + LPV/r (lopinavir/ritonavir); TDF or TAF (tenofovir alafenamide fumarate) + FTC + EVG/c (elvitegravir/cobicistat). No statistically significant difference was found between the two groups according to the drug regimens used. Table 1 summarizes the demographic and clinical characteristics of the study population.

**Table 1:** Demographic and clinical characteristics of the study population

	Total number of patients n = 225 (%)	With FMS n = 45 (%)	Without FMS n = 180 (%)	p* values
Gender				
Female	33 (14.7%)	9 (4%)	24 (10.7%)	0.258
Male	192 (85.3%)	36 (16%)	156 (69.3%)	
Median age (year)	40.52 ± 12.98	41.93 ± 11.24	40.17 ± 13.38	0.415
Marital status				
Married	84 (37.3%)	15 (6.7%)	69 (30.7%)	0.535
Single	141 (62.7%)	30 (13.3%)	111 (49.3%)	
Working status				
Working	135 (60%)	24 (10.7%)	111 (49.3%)	0.307
Not working	90 (40%)	21 (9.3%)	69 (30.7%)	
Duration of HIV infection (year)	4.74 ± 4.42	6.36 ± 5.83	4.29 ± 3.84	*0.007*
Comorbidity				
Yes	36 (16%)	12 (5.3%)	24 (10.7%)	*0.029*
No	189 (84%)	33 (14.7%)	156 (69.3%)	
Median CD4 count (cell/mm^3^)	616.0 ± 303.9	501.57 ± 309.0	642.70 ± 297.3	*0.006*
Median viral load (> 40 copy/ml)	19,867.2 ± 72,559.9	16,235.1 ± 4241.9	20,775.2 ± 78,371.9	0.708
ART regimens				
TDF + FTC + RAL	24 (10.7%)	3 (6.7%)	21 (11.7%)	0.375
TDF + FTC + DTG	17 (7.6%)	5 (11.1%)	12 (6.7%)	0.355
ABC + 3TC + DTG	28 (12.4%)	10 (22.2%)	18 (10.0%)	0.057
TDF + FTC + DRV/r	30 (13.3%)	3 (6.7%)	27 (15.0%)	0.188
TDF + FTC + LPV/r	42 (18.7%)	12 (26.7%)	30 (16.7%)	0.213
TDF (or TAF) + FTC + EVG/c	84 (37.3%)	12 (26.7%)	72 (40.0%)	0.249

*TDF* tenofovir disoproxil fumarate, *TAF* tenofovir alafenamide fumarate, *FTC* emtricitabine, *RAL* raltegravir, *DTG* dolutegravir, *ABC* abacavir, *3TC* lamivudine, *DRV/r*:darunavir/ritonavir; *LPV/r* lopinavir/ritonavir; *EVG/c* elvitegravir/cobicistat

* *p* < 0.05 was considered significant

In terms of assessment scales, a statistically significant difference was found in FIQ, BDI, PSQI, FSS and all subgroups of the SF-36 scale between the patients with and without FMS (p = 0.001) (Table 2).

**Table 2:** Assessment scales of the study population

Assessment scales	Total of patients n = 225 (%)	With FMS n = 45 (%20)	Without FMS n = 180 (%80)	p* values
FIQ	35.68 ± 22.72	58.53 ± 19.58	29.97 ± 19.69	*0.001*
BDI	16.41 ± 12.63	26.33 ± 14.51	13.93 ± 10.81	*0.001*
PSQI	6.16 ± 3.94	8.67 ± 3.87	5.53 ± 3.71	*0.001*
FSS	4.09 ± 1.82	5.49 ± 1.14	3.75 ± 1.79	*0.001*
SF-36				
Physical function (PF)	80.33 ± 27.68	52.00 ± 34.48	87.42 ± 20.36	*0.001*
Physical role (PR)	64.67 ± 39.27	36.67 ± 39.02	71.67 ± 36.17	*0.001*
Emotional role (ER)	58.67 ± 40.77	22.22 ± 31.78	67.77 ± 37.61	*0.001*
Vitality (V)	56.53 ± 25.00	32.67 ± 19.96	62.50 ± 22.47	*0.001*
Mental health (MH)	56.53 ± 22.81	44.27 ± 16.87	59.60 ± 23.11	*0.001*
Social function (SF)	69.37 ± 26.47	50.83 ± 27.49	74.00 ± 24.14	*0.001*
Bodily pain (BP)	70.03 ± 24.17	49.50 ± 27.84	75.17 ± 20.20	*0.001*
General health (GH)	55.07 ± 25.69	33.00 ± 23.34	60.58 ± 23.21	*0.001*

*FIQ* Fibromyalgia Impact Questionnaire, *BDI* Beck Depression Inventory, *PSQI* Pittsburgh Sleep Quality Index, *FSS* Fatigue Severity Scale, *SF*-*36* Short- Form 36

* *p* < 0.05 was considered significant

In the bivariate analysis of assessment scales, a statistically significant relationship was found between FIQ-PSQI, FIQ-PR, FIQ-GH, BDI-FSS, BDI-ER, BDI-V, BDI-BP and BDI-MH (Table 3).

**Table 3:** Bivariate analysis of measurement outcomes in FMS (+) patients

	Pearson coefficient of correlation (r)*	p-values (*p*)**
FIQ-PSQI	0.538	*0.001*
FIQ-PR	− 0.559	*0.001*
FIQ-GH	− 0.575	*0.001*
BDI-FSS	0.526	*0.001*
BDI-ER	− 0.519	*0.001*
BDI-V	− 0.592	*0.001*
BDI-BP	− 0.619	*0.001*
BDI-MH	− 0.604	*0.001*

*FIQ* Fibromyalgia Impact Questionnaire, *BDI* Beck Depression Inventory, *PSQI* Pittsburgh Sleep Quality Index, *FSS* Fatigue Severity Scale, *PR* physical role, *GH* general health, *ER* emotional role, *V* vitality, *BP* bodily pain, *MH* mental health

*r: Pearson coefficient of correlation

** *p* < 0.05 was considered significant

## Discussion

In our study, the prevalence of FMS as a cause of chronic widespread pain was found to be 20% in HIV/AIDS patients. The patients included in our study were taking six different ART regimens, and we could not determine an evidence of an interaction between the ART regimen and the presence of FMS. Pain negatively affects daily life activities, psychological status, social life and quality of life in HIV/AIDS patients [22]. Therefore, the effects of FMS as a cause of generalized pain on function, depression, sleep quality; fatigue and quality of life as well as the prevalence of FMS in HIV infected patients were prospectively investigated. According to the results of our study, the presence of FMS had a negative effect on function, increased depression and fatigue, and decreased sleep quality and quality of life (p = 0.001).

Presence of concomitant FMS in HIV/AIDS patients was investigated by Buskila et al. for the first time and the prevalence of FMS was found to be 29% [23]. In the following studies, prevalence of FMS ranging between 1% and 14.1% was reported [9–11, 24]. In our study, the prevalence of FMS in HIV/AIDS patients was found to be 20% which is slightly higher compared to previous studies. This discrepancy may be attributed to various factors, such as race, disease duration and differences in ART selection. However, we can say that the most important factor is the use of more up-to-date FMS diagnostic criteria in our study.

The ACR 1990 diagnostic criteria had been used for long years for the diagnosis of FMS. However, ACR 1990 diagnostic criteria could not determine the severity of disease could not be used in the follow-up and did not include the complaints of fatigue, sleep or cognitive disorder. For these reasons, ACR published the new diagnostic criteria in 2010 [25]. Another revision was made in 2016 because it was observed in time that the 2010 diagnostic criteria were insufficient in differentiation of regional pain syndromes and in detection of concomitant FMS [12]. Similar to our study, another study using the revised current diagnostic criteria in 2016 for FMS diagnosis could not be reached.

In our study, the duration of disease was longer in patients with FMS than in patients without FMS (p = 0.007). Similarly, Naorem et al. found a relationship between FMS and disease duration in patients receiving ART [10]. In another study, it was reported that the risk of rheumatologic disease increases with prolongation of the disease [5]. According to these data, even if HIV infection is controlled by ART regimens, FMS-related symptoms may occur over the years. Regardless of infection processes, FMS can develop with complex mechanisms that are not yet clearly understood. Our results determined that CD4 count found lower in patients with FMS than the ones without FMS (p = 0.006). According to international guidelines, the risk of comorbidity increases if CD4 < 200 cells/mm^3^ [26]. An increased risk of osteoarticular infections such as septic arthritis, prosthetic infections, osteomyelitis has been shown to increase if the CD4 count is < 200–400 cells/mm^3^ [27]. In our study, CD4 count > 500 cells/mm^3^ in both groups. So, CD4 count was not considered as a risk factor for comorbidity and osteoarticular involvement, nor was it considered as a risk factor for musculoskeletal involvement. The difference between the patients with FMS and without FMS was thought to be due to differences in individual immunity. On the other hand, some studies noticed any relationship between CD4 count and the presence of FMS [9, 24]. Due to conflicting results, the relationship between FMS development and CD4 count in HIV-infected patients has not yet been clearly understood.

All of the participants in our study were receiving ART treatment. When ART regimens were examined in six groups, it was seen that the treatment regimen was not associated with FMS (p > 0.05). This result was consistent with the results of other studies [9, 10, 24]. Kaddu-Mukasa et al. found a similar result and attributed the result to the low number of patients (n = 62) receiving ART treatment in the study population [6]. Unlike our study, Parperis et al. stated that ART regimens containing integrase inhibitors may be associated with rheumatic involvement [5]. The use of ritonavir and saquinavir was thought to cause atralgia. Indinavir treatment has been associated with atralgia, monoarthritis, oligoarthritis and adhesive capsulitis [28]. However, these drugs are not included in the ART regimens included in our study.

HIV infection may cause impairment in functional level. Functional impairment has been related mostly with comorbidities, low physical activity and pain [29]. In our study, it was found that the functional level was lower in the patients with FMS compared to the ones without FMS (p = 0.001). In one study, it was reported that the participants could perform most daily life activities, though they had musculoskeletal involvement [30]. This contradiction may be due to the fact that we evaluated only presence of FMS as musculoskeletal involvement and used FIQ which is a specific scale evaluating physical function and health status in patients with FMS. In our study, a significant correlation was found between FIQ and the PR and GH subscales of the SF-36 scale in patients with FMS. When functional level worsens in patients with FMS, quality of life related to physical health and self-perception of health decreases (Table 3).

Depression is the most common neuropsychiatric complication in HIV/AIDS patients. Studies have found that chronic pain coexisting HIV infection increases depressive symptoms. As the severity of chronic pain increases, the severity of depression also increases [31]. The effect of FMS, a reason for chronic pain, on depression was investigated in this study, and it was shown that the patients with FMS were more depressive compared to the patients without FMS (p = 0.001). Our result supports the study in which depression was found with a higher rate in HIV/AIDS patients with FMS compared to the ones without FMS [9].

HIV infected patients have a greater tendency to sleep disorders compared to the general population. Pain is an undeniable risk factor leading to sleep disorders. In a recent meta-analysis, sleep disorders were reported with a higher rate in HIV/AIDS patients with chronic pain compared to HIV/AIDS patients without pain [32]. Whatever its cause is, the pain is associated with poor sleep quality, low sleep efficiency and impaired sleep time [33]. The results obtained in our study are in consistent with this data. In our study, we found that FMS, which was a cause of generalized pain, had more adverse effects on sleep compared to the patients without FMS (p = 0.001). In the study by Dotan et al., insomnia was evaluated with patients’ self-reports in HIV/AIDS patients and no difference was found between the patients with and without FMS [9]. On the other part, we evaluated sleep quality with PSQI [18] which is used very commonly and has a high level of validity and reliability. Therefore, we are of the opinion that the result we reached is more utilizable. Another important point is that sleep disorders adversely affect functional level in HIV infected patients [34]. Consistent with this information, we found a significant correlation between poor sleep quality and functional impairment in the patients with FMS (p = 0.001).

One of the most common complaints is fatigue in both patients with FMS and HIV/AIDS patients. The outcome that fatigue was observed with a higher rate in HIV/AIDS patients with FMS compared to the ones without FMS in our study was consistent with the literature [9]. In HIV/AIDS patients, fatigue was associated with depression [35]. Similar to this study, we found a strong correlation between fatigue and depression in patients with FMS (p = 0.001). In other words, concomitant FMS is expected to increase the severity of fatigue in HIV infected patients.

HIV infection has currently been considered a chronic disease due to the management of successful antiretroviral therapies. Patients’ life expectancy is prolonged. Consequently, efforts to improve the quality of life in HIV/AIDS patients have increased. Negative effect of pain on quality of life has been shown in HIV/AIDS patients [22]. Inadequate pain management may also impair quality of life [36]. In two studies evaluating the quality of life with SF-36 scale, quality of life was found to be poorer in HIV/AIDS patients with musculoskeletal involvement compared to the ones without musculoskeletal involvement [11, 30]. In our study, quality of life was also evaluated with SF-36. In all subscales of SF-36, quality of life was found to be poorer in the patients with FMS compared to the ones without FMS (p = 0.001). In one study, especially fatigue and muscle/joint pain among musculoskeletal involvements were associated with poor quality of life in HIV infected patients [30]. However, we did not obtain a relationship between fatigue and quality of life in the patients with FMS. On the one hand, a strong correlation was found between depression and the ER, V, BP, and MH subscales of the SF-36 scale in the patients with FMS (p = 0.001). Presence of depression decreases quality of life by adversely affecting psychological and mental state, vitality, and pain in HIV/AIDS patients with FMS. As in our study, Coleman noticed that depressive symptoms were associated with emotional well-being and role limitations on emotional health [37]. After all, depression has negative effects on the different components of quality of life in HIV infected patients with FMS.

A strong aspect of our study is that it is one of the few studies examining the frequency of FMS, which is a musculoskeletal involvement in HIV/AIDS patients. Moreover, this is the first study to examine the presence of FMS using current diagnostic criteria and to investigate the effects of FMS on internationally accepted scales. Our study has also potential limitations. First, our results cannot be generalized because it was conducted in a single center where only Turkish patients were followed up. A relatively small sample of HIV infected patients was recruited in the study. Male patients predominated in our study and there was no homogeneity in terms of sex. This might have affected the prevalence of FMS. Another limitation is that the long-term effects of FMS could not be examined because our study was a cross-sectional study.

## Conclusion

In conclusion, a high frequency of FMS was determined in HIV/AIDS patients in our study. No relationship was found between ART regimens and FMS. It was shown that presence of FMS adversely affected the function, depression and fatigue levels, sleep and quality of life in our prospective study. These results obtained from our study are important in the follow-up of HIV/AIDS patients receiving ART. Detection of FMS may decrease depression, fatigue and sleep disorders and increase quality of life in HIV/AIDS patients. FMS should be distinguished correctly for an accurate management of HIV and for increasing ART compliance. The current ART regimens are not yet fully understood in terms of musculoskeletal involvement. Therefore, this issue can be elucidated with large multi-center studies.

## Data Availability

Not applicable.
